# Piled-up Risk Factors: a Case Report of Diogenes Syndrome

**DOI:** 10.1192/j.eurpsy.2024.277

**Published:** 2024-08-27

**Authors:** J. L. Freixo, S. G. Rodrigues, T. Novo, D. Brandão

**Affiliations:** Psychiatry, Unidade Local de Saúde do Alto Minho, Viana do Castelo, Portugal

## Abstract

**Introduction:**

Diogenes Syndrome (DS) is an uncommon neurobehavioral syndrome characterized by social isolation, extreme neglect of personal care and a tendency to excessively accumulate useless objects in the home, usually leading to unsanitary living conditions. It is further characterized by a lack of insight into the condition, leading to a refusal to seek assistance.

**Objectives:**

To outline the clinical features of primary DS, unassociated with other psychiatric conditions, emphasizing key risk factors contributing to its development.

**Methods:**

Descriptive report of a case of DS, based on an interview with the patient, review of his clinical file, and a non-systematic literature review using the PubMed database.

**Results:**

We report a case of a 62-year-old man, widowed since the age of 33, without children, living alone in a rural area in the north of Portugal. Currently retired, he worked as a Philosophy Professor. He had no known psychiatric history until 2015, when he attended two psychiatric appointments, due to anxiety and changes in sleep pattern. He has since lost psychiatric follow-up and in May 2022 he was brought to the emergency department by his neighbor, due to changes in his behavior. He was seen several times rummaging trough trash and he didn’t leave the house for a few weeks, resulting in a cluttered and unsanitary living space. He looked malnourished, unkempt and dirty. Despite not recognizing his behavior as problematic, he accepted hospitalization. No obsessive-compulsive, depressive or psychotic symptoms were detected, nor were dysfunctional personality traits. Reversible causes of dementia were excluded, a cranioencephalic CT scan revealed no abnormal findings and a neuropsychological assessment showed no changes in cognitive functions. Post-discharge, local health services provided home support, with meal delivery and house cleaning. However, he did not buy the medication and canceled the home support service several times, ending up being hospitalized again. After this second hospitalization in August 2023, he went to live with his brother in another city and has remained stable, medicated with an SSRI and low dose Aripiprazole.

**Conclusions:**

Primary Diogenes Syndrome is rare and and its etiopathogenesis remains poorly understood. It is known that there is no distinction between genders, profession or socioeconomic status, and that it is more common in the elderly, single people, widowers and people with poor or non-existent social links with their local community. Familiarity with DS characteristics enables earlier recognition of such individuals, thereby facilitating prompt provision of social and clinical support in order to reduce morbidity, mortality, and enhance public health.

**Disclosure of Interest:**

None Declared

